# Adverse reactions of vancomycin in humans: A protocol for meta-analysis

**DOI:** 10.1097/MD.0000000000022376

**Published:** 2020-09-18

**Authors:** Yang Peng, Chen-yang Li, Zhi-ling Yang, Wei Shi

**Affiliations:** a Department of Pharmacy, Hunan Provincial People's Hospital, the First Affiliated Hospital of Hunan Normal University, Changsha; b Xinjiang Institute of Materia Medica, Xinjiang, China.

**Keywords:** adverse reaction, human, meta-analysis, protocol, vancomycin

## Abstract

**Background:**

Vancomycin is effective against *Gram-positive* bacteria and considered as a last resort in the case of ineffective use of other antigens. While due to the occurrence of adverse reactions, the application of vancomycin is strictly limited. We will conduct a meta-analysis to summarize adverse reactions of vancomycin in humans.

**Methods:**

To collect comprehensive randomized controlled trials (RCTs), the following electronic databases will be searched: PubMed, Embase, Web of Science, Cochrane Library, the China National Knowledge Infrastructure, Chinese Biomedical Literature Database, and China Science and Technology Journal Database. The range of publication time will be from the inception of the database to August 2020 without language limitation. Two reviewers will independently conduct selection of studies, data extraction and management, and assessment of risk of bias. Any disagreement will be resolved by discussion with the third reviewer. Review Manager 5.3 (The Nordic Cochrane Centre, The Cochrane Collaboration) will be used for meta-analysis. The Cochrane risk of bias tool will be used to assess the risk of bias.

**Results:**

This study will synthesize the data from the present eligible high quality RCTs to explore the incidence of adverse reactions such as hypersensitivity reactions, nephrotoxicity, ototoxicity, phlebitis, and agranulocytosis.

**Conclusion:**

This meta-analysis will provide systematic evidence for adverse reactions of vancomycin in humans.

**Study registration number:**

INPLASY202080094

## 1 Introduction

Vancomycin is a glycopeptide antibiotic isolated from the fermentation broth of *Streptomyces orientalis*.^[1]^ It is effective against Gram-positive bacteria by disrupting cell wall synthesis and has been approved for clinical use for more than 60 years.^[2,3]^ It is commonly used for methicillin-resistant *Staphylococcus aureus* (MRSA), ampicillin-resistant *enterococci* and *Gram-positive* organisms in patients allergic to penicillin.^[4–6]^ Vancomycin is usually given by intravenous drip. In the treatment of Clostridium difficile-associated disease, vancomycin is taken orally.^[7,8]^ Because of the strong bactericidal effect, Vancomycin is often considered as a last resort in the case of ineffective use of other antigens.^[9]^ While due to the occurrence of adverse reactions, the application of vancomycin is strictly limited.^[10]^

The main adverse reactions of vancomycin include hypersensitivity reactions, nephrotoxicity, ototoxicity, and so on. The most common manifestations of hypersensitivity reaction are hypersensitivity macular cutaneous rashes and anaphylaxis.^[11]^ The major effects of vancomycin-induced hypersensitivity reactions are vasodilatation, bronchoconstriction, capillary permeability increase, autonomic nervous system activation, and mucosal hypersecretion.^[12,13]^ One study showed that after vancomycin intravenously, 7%–17% of patients infected with MRSA presented nephrotoxicity.^[14]^ The dose, duration, and plasma concentration of vancomycin are all closely related to the incidence of nephrotoxicity.^[15]^ Cases of hearing loss may be related to vancomycin because the drug damages auditory branch of the eighth cranial nerve directly.^[16]^ Furthermore, some minor adverse reactions such as reversible neutropenia, reversible agranulocytosis, gastrointestinal symptoms, and pseudomembranous colitis should not be ignored.^[17,18]^

Up to now, no meta-analysis has been performed on the adverse reactions of vancomycin. In view of this, we have an opportunity to evaluate adverse reactions of vancomycin in humans comprehensively. Therefore, we will conduct a meta-analysis to summarize adverse reactions of vancomycin in humans.

## 2 Methods

### 2.1 Study registration

This study has been registered on INPLASY (INPLASY202080094). This meta-analysis will be performed according the Preferred Reporting Items for Systematic Reviews and Meta-Analyses (PRISMA) statement checklist.^[19]^

### 2.2 Eligibility criteria for study selection

#### 2.2.1 Types of studies

Only randomized controlled trials (RCTs) on clinical application of vancomycin will be considered without language limitation. Case reports, reviews, non-RCTs, and animal experiments will be excluded.

#### 2.2.2 Types of participants

Participants who received vancomycin therapy will be included without restrictions of age, gender, and race.

#### 2.2.3 Types of interventions

In the experimental group, patients were given vancomycin with no limitations of administration routes, frequency, and treatment period.

In the control group, no limitations were applied to the control treatments. However, studies used the combination of vancomycin and other treatments will not be included.

#### 2.2.4 Types of outcomes

The incidence of adverse reactions (such as hypersensitivity reactions, nephrotoxicity, ototoxicity, phlebitis, and agranulocytosis) will be designated as the outcomes.

### 2.3 Search strategy

The following electronic databases will be searched: PubMed, Embase, Web of Science, Cochrane Library, the China National Knowledge Infrastructure, Chinese Biomedical Literature Database, and China Science and Technology Journal Database. The range of publication time will be from the inception of the database to August 2020 without language limitation. The detailed search strategy of PubMed is shown in Table 1. The similar search strategies will be used for other electronic databases.

**Table 1:**
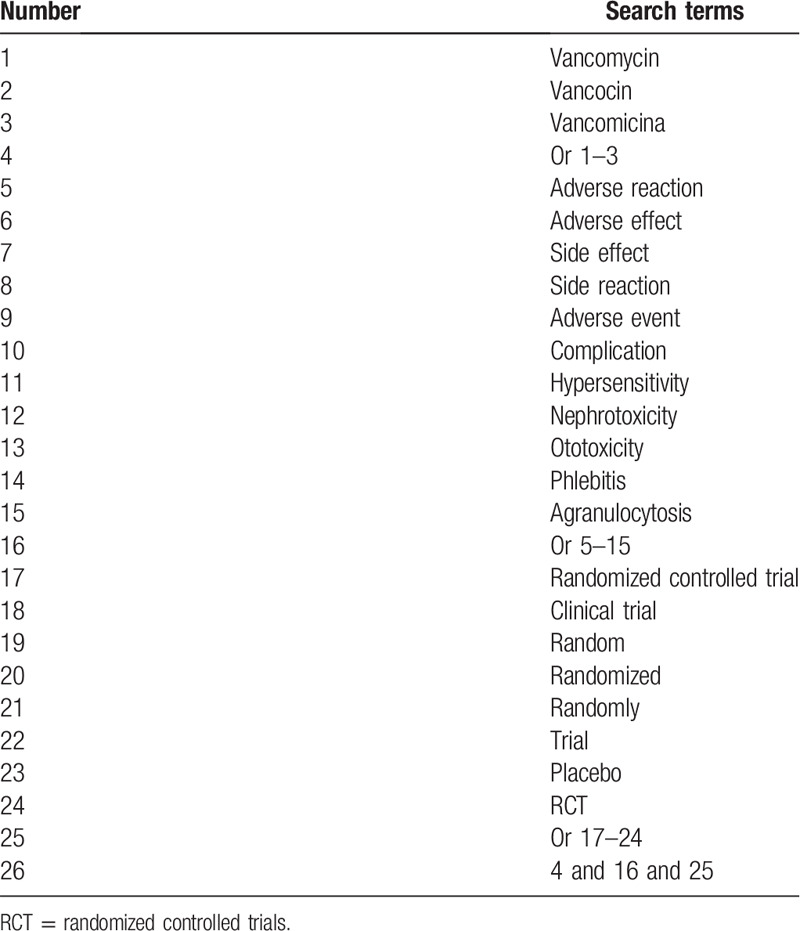
Search strategy of PubMed.

### 2.4 Selection of studies

EndNote 7.0 (Thomson Reuters) will be used to manage all retrieved citations. After removing duplicates, 2 reviewers will independently scan titles and abstracts to eliminate all irrelevant records. Then, the remaining records will be read by full texts in further assessing the inclusion of the study. Any confusion over inclusion criteria will be resolved by discussion with the third reviewer. A PRISMA flow diagram will be designed to illustrate the details of study selection.

### 2.5 Data extraction and management

After selection, 2 reviewers will independently conduct data extraction. Any confusion will be resolved by discussion with the third reviewer. If some important information is missing, we will contact original authors by email to request detailed information about the research. The general information will be extracted, including first author's name, country of publication, year of publication, title of journal, study design, patient information, experimental, and control intervention (drug names, administration routes, dose, frequency, and treatment period), and specific details about adverse events (symptoms and number of persons experiencing an adverse reaction).

### 2.6 Assessment of risk of bias

Risk of bias of the selected studies will be assessed by the Cochrane risk of bias assessment tool. This tool covers 7 aspects: random sequence generation, allocation concealment, blinding of participants and personnel, blinding of outcome assessment, incomplete outcome data, selective reporting, and other bias. A bias value of “high”, “unclear”, or “low” was given for each item. These 7 items were assessed independently by 2 reviews. Any divergences will be resolved by discussion with the third reviewer.

### 2.7 Data synthesis and analysis

#### 2.7.1 Data synthesis

Review Manager 5.3 (The Nordic Cochrane Centre, The Cochrane Collaboration) will be used for data synthesis. Odds ratio will be used for dichotomous outcomes with 95% confidence interval. Heterogeneity will be examined using the I^2^ test. The I^2^ value > 50% means significant heterogeneity, and the random effects model will be used. Otherwise, the I^2^ value ≤ 50% means minor heterogeneity, and the fixed effects model will be utilized. If significant heterogeneity still exists after subgroup analysis, meta-analysis will not be pooled, and descriptive summary will be reported.

#### 2.7.2 Subgroup analysis

Subgroup analysis will be performed to check the potential heterogeneity and inconsistency based on the different participant characteristics, administration routes, and dose of vancomycin, control methods, and outcome measurements.

#### 2.7.3 Sensitivity analysis

Sensitivity analysis will be conducted to check the robustness and reliability of pooled outcome results by excluding low-quality studies and small studies.

#### 2.7.4 Reporting bias

Publication bias will be assessed with funnel plot and Egger regression test if sufficient trials (≥10 trials) are included.^[20,21]^

## 3 Discussion

To our knowledge, this is the first meta-analysis to conduct a comprehensive literature search and provide a systematic synthesis of current published data to summarize adverse reactions of vancomycin in humans. We will search 7 electronic literature databases to avoid missing any potential eligible studies, and apply rigorous methodology to examine studies reporting the adverse reaction outcomes of vancomycin for patients. The results of this study will provide helpful evidence for clinical practice and patients, future research, as well health related policy makers.

## Author contributions

**Conceptualization:** Yang Peng, Chen-yang Li, Zhi-ling Yang.

**Data curation:** Yang Peng, Zhi-ling Yang.

**Formal analysis:** Yang Peng, Chen-yang Li, Zhi-ling Yang, Wei Shi.

**Funding acquisition:** Chen-yang Li.

**Investigation:** Chen-yang Li, Wei Shi.

**Methodology:** Yang Peng, Chen-yang Li.

**Project administration:** Zhi-ling Yang.

**Resources:** Yang Peng, Chen-yang Li, Zhi-ling Yang.

**Software:** Zhi-ling Yang, Wei Shi.

**Supervision:** Yang Peng, Zhi-ling Yang.

**Validation:** Chen-yang Li.

**Visualization:** Zhi-ling Yang, Wei Shi.

**Writing – original draft:** Yang Peng, Chen-yang Li.

**Writing – review & editing:** Zhi-ling Yang, Wei Shi.

## Abbreviations

Abbreviations: MRSA = methicillin-resistant *Staphylococcus aureus*, PRISMA = Preferred Reporting Items for Systematic Reviews and Meta-Analyses, RCTs = randomized controlled trials.
